# Visual and Proprioceptive Cue Weighting in Children with Developmental Coordination Disorder, Autism Spectrum Disorder and Typical Development

**DOI:** 10.1068/ig10

**Published:** 2013-10-01

**Authors:** L Miller, R D McIntosh

**Affiliations:** University of Edinburgh, UK; University of Edinburgh, UK

## Abstract

Accurate movement of the body and the perception of the body's position in space usually rely on both visual and proprioceptive cues. These cues are weighted differently depending on task, visual conditions and neurological factors. Children with Developmental Coordination Disorder (DCD) and often also children with Autism Spectrum Disorder (ASD) have movement deficits, and there is evidence that cue weightings may differ between these groups. It is often reported that ASD is linked to an increased reliance on proprioceptive information at the expense of visual information (Haswell et al, 2009; Gepner et al, 1995). The inverse appears to be true for DCD (Wann et al, 1998; Biancotto et al, 2011). I will report experiments comparing, for the first time, relative weightings of visual and proprioceptive information in children aged 8-14 with ASD, DCD and typical development. Children completed the Movement Assessment Battery for Children (MABC-II) to assess motor ability and a visual-proprioceptive matching task to assess relative cue weighting. Results from the movement battery provided evidence for movement deficits in ASD similar to those in DCD. Cue weightings in the matching task did not differentiate the clinical groups, however those children with ASD with relatively spared movement skills tended to weight visual cues less heavily than those with DCD-like movement deficits. These findings will be discussed with reference to previous DSM-IV diagnostic criteria and also relevant revisions in the DSM-V.

